# Short-Term Effects of a Web-Based Guided Self-Help Intervention for Employees With Depressive Symptoms: Randomized Controlled Trial

**DOI:** 10.2196/jmir.3185

**Published:** 2014-05-06

**Authors:** Anna S Geraedts, Annet M Kleiboer, Noortje M Wiezer, Willem van Mechelen, Pim Cuijpers

**Affiliations:** ^1^VU University AmsterdamDepartment of Clinical PsychologyAmsterdamNetherlands; ^2^EMGO+ Institute for Health and Care ResearchVU University Amsterdam and VU University Medical Center AmsterdamAmsterdamNetherlands; ^3^Body@Work Research Center Physical Activity, Work and HealthTNO-VU-VUmcAmsterdamNetherlands; ^4^TNOHoofddorpNetherlands; ^5^VU University Medical CenterDepartment of Public and Occupational HealthAmsterdamNetherlands

**Keywords:** depression, employees, occupational therapy, Internet, prevention

## Abstract

**Background:**

Depressive disorders are highly prevalent in the working population and are associated with excessive costs. The evidence for effective worker-directed interventions for employees with depressive symptoms is limited. Treating employees with depressive symptoms before sick leave via the Internet could be beneficial and cost saving.

**Objective:**

In this study, we developed and tested the effectiveness of a Web-based guided self-help course for employees with depressive symptoms. We report on the posttreatment effectiveness of the intervention.

**Methods:**

This study is a two-arm randomized controlled trial comparing a Web-based guided self-help course to care as usual (CAU). We recruited employees from 6 different companies via the companies’ intranet and posters. The main inclusion criterion was elevated depressive symptoms as measured by a score of ≥16 on the Center for Epidemiological Studies Depression scale (CES-D). The intervention (Happy@Work) was based on problem-solving treatment and cognitive therapy and consisted of 6 weekly lessons. Participants were asked to submit their weekly assignment via the website after completion. They subsequently received feedback from a coach via the website. Self-report questionnaires on depressive symptoms (CES-D; primary outcome), anxiety measured by the Hospital Anxiety and Depression Scale (HADS), burnout measured by the Maslach Burnout Inventory (MBI), and work performance measured by the Health and Work Performance Questionnaire (HPQ; secondary outcomes) were completed at baseline and at posttreatment.

**Results:**

A total of 231 employees were randomized to either the intervention group (n=116) or CAU (n=115).The posttreatment assessment was completed by 171 (74.0%) participants. Both the intervention and the CAU group showed significant improvements in the primary outcome of depressive symptoms, but no differences between the conditions was found (*d*=0.16, 95% CI –0.10 to 0.41, *P*=.29). Significant but small effects in favor of the intervention group were found for anxiety symptoms (*d*=0.16, 95% CI –0.09 to 0.42, *P*=.04) and exhaustion (*d*=0.17, 95% CI –0.09 to 0.43, *P*=.02).

**Conclusions:**

This study showed that a Web-based guided self-help course for employees with depressive symptoms was not more effective in reducing depressive symptoms among employees than CAU. Large improvements in depressive symptoms in the CAU group were unforeseen and potential explanations are discussed.

## Introduction

Depressive disorders are highly prevalent in the general [1-3] and working [4,5] population and lead to excessive costs [6,7]. Approximately 70%-85% of the costs are due to work absenteeism, work impairment, and loss of work productivity, which implies that companies pay the largest part of the total costs of depression [8-12]. In a recent Dutch cohort study, the total annual costs of work absenteeism due to depressive disorders were estimated at €242 million per 1 million employees, which equals €1.8 billion for the entire Dutch working population [8].

Research on the treatment of depression has been extensive [13-16] and many studies have shown positive effects of different psychotherapies (eg, [15,17,18]). Traditionally, most types of psychotherapies are delivered face-to-face in mental health care settings. However, there is increasing evidence for the effectiveness of guided self-help treatments that are delivered via the Internet [19-21]. Web-based treatments generally use the same techniques as face-to-face treatments, but patients can work through the treatment on their own in an often highly structured way. Web-based treatments may reduce costs and can increase efficiency in mental health care because of several advantages, such as high accessibility, no waiting list, and minimal contact with a professional therapist [22]. High accessibility may be of special benefit to employees because they will not lose work hours due to therapist visits outside the workplace and participation in Web-based treatments is more anonymous compared to face-to-face treatment.

The evidence for effective worker-directed interventions for employees with depressive symptoms is scarce [23]. Some research has been conducted on the treatment of employees who are absent from work (sick-listed employees) due to common mental health disorders, and the results of these studies are conflicting [23,24-27]. Previous research also shows that only a small percentage of employees with severe mental health problems seek professional treatment [28]. However, work-related aspects play an important role in the development and perpetuation of depression [23,29]. Work-related aspects, such as high job insecurity, can have a negative effect on symptom severity, and symptom severity can have a negative effect on work elements, such as job performance. Therefore, it is important to develop evidence-based worker-directed interventions for employees with depression that involve work-related aspects and the employability of the employee [23].

Recently, the Organisation for Economic Co-operation and Development (OECD) [4] published a report in which they concluded that employees with mental health problems are frequently treated when symptoms have become severe and that the work setting of the employee is not often discussed in treatment. They recommended to increase evidence-based workplace treatments for employees with mental health problems and to intervene in an earlier stage, preferably before sick leave. Other research further subscribes the importance of intervening before sick leave because it will prevent worsening of mental health problems and, as a result, it will reduce the costs of work absenteeism and loss of work productivity [29,30]. Unfortunately, there is almost no research available on the treatment of employees with mental health problems who are not on sick leave [31]. The study by Lexis and colleagues [31] showed positive results of a face-to-face problem-solving treatment for employees with a high risk for sick leave due to depressive symptoms. The promising results of this study indicate the importance of investment in intervening before sick leave. Providing such a preventive intervention via the Internet can have many advantages as previously mentioned.

The current randomized controlled trial evaluated the effectiveness of a Web-based guided self-help course for employees with depressive symptoms who were not on sick leave compared to care as usual (CAU). The intervention is aimed at reducing the employee’s depressive symptoms, and we postulate that this may reduce work absenteeism and loss of work productivity as well, which will result in cost savings for the employer. The first aim of this study was to examine whether the Web-based intervention had a more positive effect on depressive symptoms compared to the CAU group. The second aim was to investigate if the intervention had positive effects on symptoms of anxiety, burnout, and work performance.

## Methods

### Recruitment of Participants

Participants were recruited via 6 different (international) companies in the Netherlands: 2 banking companies (company 1 and 2), 2 research institutes (company 3 and 4), 1 security company (company 5), and 1 university (company 6). Participants were recruited via banners and digital pamphlets on the companies’ intranet or via posters (only in company 5). Recruitment took place between September 2011 and December 2012. Participants who were interested in taking part in the study could ask for more information about the study via email. When information was requested, one of the researchers sent an information leaflet and an informed consent form via email. The informed consent could be returned via post or email. After participants gave informed consent, they received a link to an online screening questionnaire via email. The study protocol, information leaflet, and informed consent form were approved by the Medical Ethics Committee of the VU University Medical Center (registration number 2011/2).

Participants were eligible to take part if they were 18 years of age or older, had elevated depressive symptoms as measured by a score of 16 or higher on the Center for Epidemiologic Studies Depression scale (CES-D), were not on partial or full sick leave, had access to the Internet and an email address, and were employed by 1 of the 6 participating companies. Exclusion criteria were unstable (<1 month) medication use for depressive symptoms and having a legal labor dispute with the employer.

### Procedure

#### Design

This study was a randomized controlled trial with 2 arms: a Web-based guided self-help course called Happy@Work and a CAU group. The full design of the study has been described in detail elsewhere [32].

#### Sample Size

The sample size was guided by the expected difference in the primary outcome (ie, depressive symptoms) between the 2 groups. Based on a power of 0.80, an alpha of.05, and an expected dropout percentage of 30%, we would need 100 participants in each condition to be able to show an effect-size Cohen’s *d* of 0.50. Therefore, the total sample size was determined at 200.

#### Randomization

Randomization took place at an individual level after completion of the baseline measurement (questionnaire and clinical interview). We used stratification at 2 levels: (1) use of antidepressants and (2) receiving treatment from a psychologist or psychiatrist at study entrance. Block randomization was used with random blocks containing 4, 6, or 8 allocations. An independent researcher made the allocation schedule with a computerized random number generator and the investigators had no knowledge of the schedule. Participants were randomized into 2 groups: the intervention group or the CAU group. Participants were informed about the randomization outcome via email.

### Interventions

#### Happy@Work

**Figure 1:**
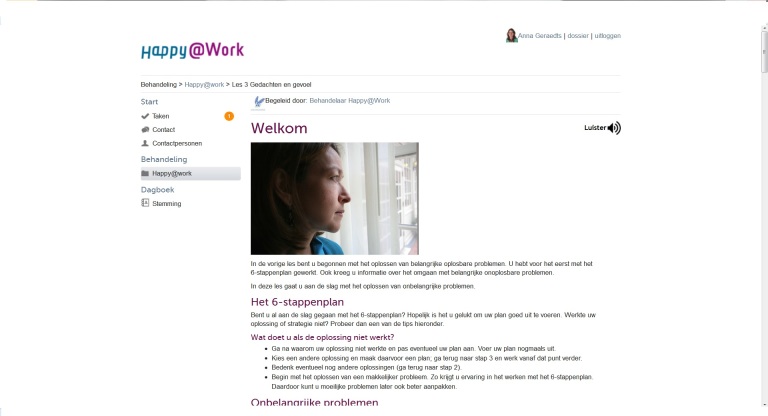
Screenshot of Happy@Work intervention.

The intervention Happy@Work [33] is a brief Web-based intervention delivered with minimal guidance. It consists of 2 evidence-based treatments: problem-solving treatment (PST) [34], cognitive therapy [35], and a guideline for employees to help them to prevent work-related stress [36,37]. In PST, it is assumed that depressive symptoms can be caused by practical problems that people face in their daily lives. It is believed that when people can resolve their problems, their symptoms of depression will decrease [38]. The PST will help them solve their problems. Sometimes, however, problem solving can be disrupted by automatic thoughts such as “I am too weak to solve this problem” or “I will fail solving this problem.” PST may not be sufficient to change these automatic thoughts that disrupt problem solving. Therefore, we incorporated cognitive therapy information and assignments to change these automatic thoughts in the course [35]. Some of the problems that people face are likely to be work-related. These problems are sometimes more difficult for people to comprehend [36,37]. Therefore, one lesson is focused on work-related problems specifically. Happy@Work primarily focuses on the depressive symptoms of the employee, but also incorporates psychoeducation and assignments related to dealing with stress and burnout symptoms because there is a clear relationship between the different constructs [29,39-42]. The intervention consists of 6 weekly lessons with an option of 1 week extra time in case of delay. Each lesson has a different theme, but always follows the same structure: information about the theme, examples, and assignments. Themes of the lessons are introduction of problem solving (lesson 1), problem-solving methods (lesson 2), changing cognitions (lesson 3), dealing with work-related problems (lesson 4), social support (lesson 5), and relapse prevention (lesson 6). Screenshots of the intervention can be found in Figure 1 and Multimedia Appendix 1.

At the start of the intervention, an account was generated by the researchers on the website and a coach was assigned to the participant on the website. Once the account was generated, an automatic email was sent to the participant with a link to activate the account. Participants used their email address and a self-created password to log in once the account was activated.

Participants were asked to submit their weekly assignment via the website after completion and subsequently they received feedback from a coach, again via the website, within 3 working days. Next, automatic emails were sent to participants to notify them about the feedback, to describe the following lesson, and to give the deadline for completion of the next assignment. Participants were able to start with a new lesson after they had received the feedback (ie tunneled intervention). When deadlines were not met, email reminders were sent to participants. There were no content changes, bug fixes, or periods of downtime required during the trial.

The coaches were Master’s students in clinical psychology with training of 6 hours. All coaches used a protocol-treatment manual throughout the course. To ensure treatment fidelity, all feedback was reviewed by a supervisor (AG) before it was placed on the website. The support includes feedback on the assignments and motivational and empathic strategies to keep participants engaged in the course. Development of a patient-therapist alliance, as in traditional psychotherapy, was not an aim of the support.

#### Care as Usual

Participants in the CAU group did not receive treatment or support from the coaches. However, in the email with the randomization outcome they were advised to consult their (occupational) physician or a psychologist if they wanted treatment for their depressive symptoms. Participants who were interested were sent a copy of the self-help book version of the intervention after having completed the posttreatment assessment. Participants in both conditions had access to any additional (mental) health care.

### Measures

#### Outcome Measures

Participants filled in several questionnaires at baseline (T0) and 8 weeks later (T1). Both assessments took place online. Participants also participated in a clinical interview at T0 via telephone.

#### Depressive Symptoms

Symptoms of depression were measured with the Center for Epidemiological Studies Depression scale (CES-D) [43]. This questionnaire is widely used for identifying people with depressive symptoms. Its validity has been tested in different populations [44-46]. The CES-D consists of 20 items and the total score varies between 0 and 60. The Cronbach alpha in this study was .82. A score of 16 or higher represents a clinically significant level of depressive symptoms [43]. The cut-off score of 16 was used in this study as an inclusion criterion.

#### Anxiety Symptoms

The anxiety subscale of the Hospital Anxiety and Depression Scale (HADS) was used to measure anxiety symptoms [47]. The anxiety subscale of the HADS consists of 7 items. Scores range from 0 to 21, with higher scores indicating more anxiety. The HADS has shown good homogeneity and reliability in different normal and clinical Dutch samples [48]. The Cronbach alpha in this study was .76.

#### Burnout Symptoms

Burnout symptoms were measured with the Dutch version of the Maslach Burnout Inventory-General Scale (MBI) [49,50]. This self-report questionnaire contains 15 items and 3 dimensions: exhaustion (5 items), cynicism (4 items), and reduced professional efficacy (6 items). Every item was scored on a 7-point Likert scale (0-6). Following the manual of the questionnaire [50], a total score for every dimension was calculated by adding the item scores and dividing that total score by the number of items, with higher scores indicating more severe symptoms. Participants with a high score on exhaustion and a high score on cynicism or a high score on reduced professional efficacy were considered as “burnout” [50]. We rescored the professional efficacy dimension with higher scores indicating less feeling of professional efficacy, hence the high score in the burnout diagnoses. The Cronbach alphas for the different dimensions in this study were .83 for exhaustion, .83 for cynicism, and .79 for reduced professional efficacy.

#### Work Performance

We used the general work performance scale of the World Health Organization (WHO) Health and Work Performance Questionnaire (HPQ) [51], which contains 4 items. Item 4 gives the best and easiest indication of the subject’s perception of their own work performance [52] and we report that item only in this study. On item 4, participants were asked to rate their overall work performance during the past 4 weeks when compared to employees with comparable functions. It was scored on a 7-point Likert scale with a higher score indicating poorer work performance compared to other employees [52].

#### Clinical Interview

The WHO Composite International Diagnostic Interview version 2.1 (CIDI) [53] is a structured interview to assess psychiatric diagnosis defined in the American Psychiatric Association’s *Diagnostic and Statistical Manual of Mental Disorders*, 4th edition, Text Revision (*DSM-IV-TR*) [54]. For this study, 2 sections of the CIDI were assessed: the mood disorders section and the “other” anxiety disorders (social phobia, panic disorder, agoraphobia, and generalized anxiety disorder) section. The CIDI was conducted by trained interviewers via telephone at baseline (T0) and was used for diagnostic purposes.

#### Health Care Utilization

A revised version of the Trimbos and iMTA Questionnaire on Costs Associated with Psychiatric Illness (TiC-P) [55] was used to collect data on health care utilization. The TiC-P is a self-report questionnaire and consists of 2 different parts that can be administrated separately. Part I was used and contains 12 items concerning health care utilization by participants. There were 2 questions added to the questionnaire on the frequency of utilization of different health care services of the company: occupational physician and occupational social work. The questionnaire was used at T0 to assess health care utilization up to 3 months before the start of the study and at posttreatment (T1) assessment to assess health care utilization between baseline and posttreatment assessment.

#### Other Measures

We added demographic questions, working hours, medication use for psychological problems, and treatment by a mental health specialist to the baseline questionnaire.

### Statistical Analysis

#### Baseline Differences

Baseline differences in demographic variables and outcome measures were investigated using chi-square tests and independent-sample *t* tests.

#### Missing Values

Baseline data were available for all participants. Missing values at posttreatment (T1) (26.0%, 171/231) were handled by multiple imputation using the fully conditioned specified method with model type predictive mean matching in SPSS version 20.0 (IBM Corp, Armonk, NY, USA) creating 5 datasets. In multiple imputation, missing data are imputed by regression analyses and the available baseline data (demographics and baseline scores on outcome measurements) of the study completers as well as the study dropouts at posttreatment are used to estimate missing values at posttreatment. This provides a more reliable estimation of the “real” data than other imputation methods. The analyses were performed on the 5 created datasets and combined into a single overall estimate using the multiple imputation inference rules of Rubin [56]. This yields proper *P* values and confidence intervals for the estimates.

#### Effectiveness

Primary effectiveness analyses were conducted according to the intention-to-treat (ITT) principle. We calculated the intraclass correlation (ICC) to examine nonindependence of observations at the company level. The results showed that this was not an issue (ICC=.02, *P*=.77); therefore, a multilevel approach for analyzing the data was not deemed necessary. We performed linear regression analyses to determine differences between the intervention group and the CAU group with the posttreatment score as the dependent variable and group (intervention or CAU) as the predictor variable while controlling for baseline scores for every outcome measure. The magnitude of the effect is expressed in Cohen’s *d* [57]. The Cohen’s *d* was calculated by subtracting the post treatment mean score of the CAU group of the posttreatment mean score of the intervention group and dividing that result by the pooled standard deviation. Effect sizes of ≥0.8 are assumed to be large, effect sizes of 0.5-0.8 are moderate, and effect sizes of 0.2-0.5 are assumed to be small [57]. We calculated the effect sizes for all participants (ITT) and for participants who completed the intervention (per protocol analysis). Completion of the intervention was defined as completion of ≥5 lessons of the intervention. Reporting both outcomes is of high importance because intervention dropout is common in Web-based interventions.

We calculated clinical significant change as described by Jacobson and Truax [58]. This method uses a reliable change index as an index for improvement and its formula is pretest score minus posttest score divided by the standard error of the difference between the 2 test scores. To calculate the standard error of the difference between the 2 test scores in the formula, one uses the pretest SDs of the outcome and the reliability of the questionnaire. In this study, we used the following reliability scores from the questionnaires: 0.90 for the CES-D [59], 0.83 for the HADS [60], 0.88 for the exhaustion scale of MBI [50], 0.81 for the cynicism scale of MBI [50], and 0.75 for the reduced professional efficacy scale of MBI [50]. If the outcome of the sum of the reliable change index is greater than 1.96, this is considered a significant improvement because the amount of change is unlikely to have occurred by chance. The differences in clinical significant improvement rates were then expressed as odds ratios (ORs).

We calculated the recovery rates for depressive symptoms in which a score <16 on the CES-D at posttreatment was defined as recovery [59]. The differences in recovery rates were then expressed as ORs. Finally, we defined reliable recovery as clinical significant improvement between baseline and posttreatment score and recovery at posttreatment. Reliable recovery was also expressed as OR. Results are presented as the mean and standard deviations of the observed data. Between- and within-group effect sizes as well as effectiveness analyses were based on the pooled results of the imputed data.

## Results

### Participants and Response Rates

#### Overview

**Figure 2:**
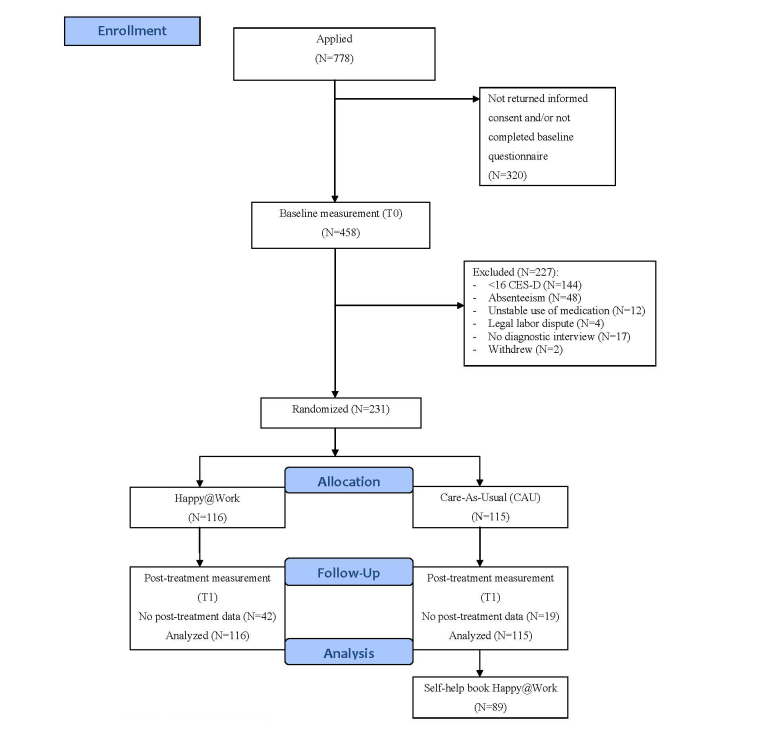
Flowchart of participants.

Figure 2 shows the flow of participants through the trial. A total of 778 employees were interested in the study and applied for more information; 320 of them did not return the informed consent form and/or did not fill in the baseline questionnaire. We received a completed informed consent form and a baseline questionnaire from 458 participants. Of those, 208 were not eligible to take part in the study because they scored below the cut-off score of 16 on the CES-D (n=144), were already absent from work (n=48), had unstable medication use for depressive symptoms (n=12), or had a legal labor dispute with their employer (n=4). Further, 17 participants did not complete the diagnostic interview and 2 participants withdrew from the study before randomization. The remaining 231 participants were randomized to either the intervention group (n=116) or the CAU group (n=115). Most participants (n=166) were employed by 1 of the 2 banking companies, 39 by the research institutes, 11 by the security company, and 15 by the university. Of the 231 participants, 10 (4.3%) used medication without psychological treatment, 24 (10.4%) received psychological treatment but no medication, and 4 participants (1.7%) used both medication and received psychological treatment at baseline. Thus, most participants (83.6%) did not receive treatment for their depressive symptoms.

As shown in Table 1, most participants were female (62.3%, 144/231), born in the Netherlands (95.2%, 220/231), involved in an intimate relationship (76.2%, 176/231), highly educated (63.6%, 147/231), and worked for 34 hours per week on average. Participants in the intervention group were more often born outside the Netherlands (7.8%, 9/116) than participants in the CAU group (1.7%, 2/115, *P*=.03). There were no significant differences between the intervention group and the CAU group on any of the outcome measures at baseline.

**Table 1:** Participants’ demographic characteristics at baseline.

Characteristic		All (N=231)	Intervention (n=116)	CAU (n=115)	*P* value
Age (years), mean (SD)		43.4 (9.2)	43 (8.9)	43.8 (9.6)	.51
**Gender, n (%)**					.20
	Female	144 (62.3)	77 (66.4)	67 (58.3)	
	Male	87 (37.7)	39 (33.6)	48 (41.7)	
**Country of birth, n (%)**					.03
	Netherlands	220 (95.2)	107(92.2)	113 (98.3)	
	Other	11 (4.8)	9 (7.8)	2 (1.7)	
**Marital status, n (%)**					.46
	Relationship	176 (76.2)	86 (74.1)	90 (78.3)	
	No relationship	55 (23.8)	30 (25.9)	25 (21.7)	
**Education,** ^a^ **n (%)**					.25
	Low	16 (6.9)	11 (9.5)	5 (4.3)	
	Middle	68 (29.4)	31 (26.7)	37 (32.2)	
	High	147 (63.6)	74 (63.8)	73 (63.5)	
Working hours,^b^ mean (SD)		33.9 (5.0)	33.7 (4.8)	34.0 (5.3)	.65
Working days, mean (SD)		4.3 (0.7)	4.3 (0.6)	4.2 (0.7)	.32

^a^Low: primary education or lower general secondary education, middle: intermediate vocational education or high school, high: higher vocational education or university.

^b^Mean working hours per week according to contract of the employee.

#### Diagnosis

A total of 57 participants (24.7%) were diagnosed with a current major depressive disorder, dysthymic disorder, or both; 23 participants in the intervention group and 34 in the CAU group. Of those, 15 participants suffered from a single episode major depressive disorder (9 intervention, 6 CAU), 40 participants from a recurrent major depressive disorder (12 intervention, 28 CAU), and 9 participants from a dysthymic disorder (7 intervention, 2 CAU).

Anxiety disorders were less frequently present; a total of 48 participants (20.8%) were diagnosed, 27 participants in the intervention group and 21 in the CAU group. The different anxiety disorders frequencies were as follow: social phobia (14 intervention, 7 CAU), panic disorder without agoraphobia (2 intervention, 0 CAU), panic disorder with agoraphobia (4 intervention, 2 CAU), or generalized anxiety (11 intervention, 16 CAU) disorder.

#### Health Care Utilization

At posttreatment, we analyzed the health care utilization of both groups to get a more detailed view on health care utilization by the CAU group. Only a small number of the total participants made use of health care. More participants in the CAU group sought help in general health care (19 intervention, 25 CAU) or occupational health care (4 intervention, 7 CAU), but this was not statistically significant compared to the intervention group (*t*_43_=0.35, *P*=.73; *t*_9_=1.09, *P*=.31). There was only a slight difference in medication use for depressive symptoms between the groups (8 intervention, 10 CAU), and this was not statistically significant different (χ^2^_1_=0.00, *P*=.96).

### Attrition and Adherence

#### Study Attrition

A total of 61 participants (26.4%) did not complete the T1 (posttreatment) assessment: 42/116 (36.2%) of the intervention group and 19/115 (16.5%) of the CAU group. Data from 173 of 231 participants (74.9%) was available for the primary outcome depressive symptoms, and data from 171of 231 participants (74.0%) was available for the other outcomes. Participants in the CAU group more often completed the T1 assessment (χ^2^_1_=11.5, *P*=.001). Attrition rates were also lower for participants who completed the intervention (χ^2^_1_=32.1, *P*<.001).

#### Treatment Adherence

Of the participants randomized to the intervention group, 9.5% (11/116) did not start or complete the first lesson of Happy@Work. Lesson 1 was completed by 90.5% (105/116), lesson 2 by 75% (87/116), lesson 3 by 57.8% (67/116), lesson 4 by 49.1% (57/116), lesson 5 by 38.8% (45/116), and lesson 6 by 26.7% (32/116). A total 29 of 116 participants dropped out of the intervention, the other participants were not able to complete more lessons within the time limit of 7 weeks. Most participants who dropped out did not report a reason for dropout. When reasons were reported (14/116) they pertained mostly to lack of time.

### Effects

#### Improvements on Outcome Measures

All participants improved between baseline and posttreatment on all outcomes measured (see Table 2). For the primary outcome, depressive symptoms, a high within-group effect-size Cohen’s *d* was found for both the intervention (*d*=1.03, 95% CI 0.76-1.30, *P*=.001) and the CAU group (*d*=0.98, 95% CI 0.71-1.25, *P*<.001). However, there was no difference between both groups (*d*=0.16, 95% CI –0.10 to 0.41, *P*=.29). The same result was found when we compared course completers (n=45) with CAU (*d*=0.29, 95% CI –0.05 to 0.64, *P*=.13).

Small to medium within-group effects were found for the secondary outcomes anxiety, burnout, and work performance in both the intervention group and the CAU group (see Table 2). Between-group differences were small for all secondary outcomes, but participants in the intervention group improved significantly more on anxiety symptoms (*d*=0.16, 95% CI –0.09 to 0.42, *P*=.04) and the exhaustion dimension of the MBI (*d*=0.17, 95% CI –0.09 to 0.43, *P*=.02) compared to CAU. Course completers also improved more on anxiety symptoms compared to CAU (*d*=0.19, 95% CI –0.16 to 0.53, *P*=.04), but not on the exhaustion dimension of the MBI (*d*=0.17, 95% CI –0.18 to 0.52, *P*=.14). No significant between-group differences were found on the cynicism dimension of the MBI, the reduced professional efficacy dimension of the MBI, and work performance.

**Table 2:** Effects of intervention (n=116) versus care-as-usual (n=115) group with course completers (CC).

Outcome		Pretest, mean (SD)	Posttest, mean (SD)	Effect size,^a^ Cohen’s *d* (95% CI)		
				Within	Between (all)	Between (CC, n=45)
**CES-D**					0.16 (–0.10, 0.41)	0.29 (–0.05, 0.64)
	Intervention	25.7 (7.5)	15.8 (10.6)	1.03 (0.76, 1.30)^b^		
	CAU	26.1 (7.0)	18.3 (9.1)	0.98 (0.71, 1.25)^c^		
	Intervention-CC	25.3 (6.5)	15.1 (10.4)			
**HADS**					0.16 (–0.09, 0.42)^d^	0.19 (–0.16, 0.53)^d^
	Intervention	10.6 (3.8)	7.6 (3.8)	0.77 (0.51, 1.04)^c^		
	CAU	10.2 (3.2)	8.3 (3.6)	0.56 (0.29, 0.82)^c^		
	Intervention-CC	10.7 (3.6)	7.5 (4.0)			
**MBI-exhaustion**					0.17 (–0.09, 0.43)^e^	0.17 (–0.18, 0.52)
	Intervention	3.3 (1.2)	2.7 (1.2)	0.50 (0.24, 0.76)^c^		
	CAU	3.3 (1.1)	3.0 (1.2)	0.35 (0.09, 0.61)^c^		
	Intervention-CC	3.3 (1.2)	2.7 (1.1)			
**MBI-cynicism**					0.30 (0.05, 0.57)	0.31 (–0.04, 0.65)
	Intervention	2.8 (1.3)	2.4 (1.3)	0.31 (0.05, 0.57)^c^		
	CAU	3.1 (1.3)	2.8 (1.3)	0.23 (-0.03, 0.49)^c^		
	Intervention-CC	2.7 (1.2)	2.4 (1.3)			
**MBI-reduced professional efficacy**					0.10 (–0.16, 0.36)	0.30 (–0.05, 0.65)
	Intervention	2.6 (1.0)	2.4 (1.0)	0.20 (-0.06, 0.46)^c^		
	CAU	2.7 (0.9)	2.5 (0.9)	0.21 (-0.05, 0.47)^c^		
	Intervention-CC	2.4 (1.0)	2.2 (1.0)			
**HPQ-4**					0.00 (–0.26, 0.26)	0.07 (–0.28, 0.41)
	Intervention	4.1 (1.6)	3.6 (1.5)	0.32 (0.06, 0.58)^c^		
	CAU	4.3 (1.8)	3.6 (1.5)	0.42 (0.16, 0.68)^c^		
	Intervention-CC	4.3 (1.7)	3.4 (1.5)			

^a^The effect size is presented as Cohen’s *d*: the number of standard deviations in the intervention group has improved more than the CAU group; (CAU mean–intervention mean)/pooled SD.

^b^ *P*=.001.

^c^ *P*<.001.

^d^ *P*=.04.

^e^ *P*=.02.

#### Clinical Significant Improvement and Reliable Recovery

Data on clinical significant improvement are reported in Table 3. Clinical significant improvement on depressive symptoms was comparable in both groups (OR 0.9, 95% CI 0.5-1.6, *P*=.82). More participants in the intervention group showed clinical significant improvement on anxiety symptoms, the exhaustion dimension of the MBI, and the cynicism dimension of the MBI compared to the CAU group, but differences between the groups were not significant (see Table 3). A total of 105 of the 231 participants (45.5%) were recovered from depression at posttreatment. More participants in the intervention group (56/116, 48.3%) recovered from depression compared to the CAU group (49/115, 42.6%), but not significantly (OR 1.3, 95% CI 0.7-2.3, *P*=.41). Reliable recovery rates for depression were also in favor for the intervention group, with an odds ratio of 1.3. In the intervention group, 44.8% (52/116) reliably recovered and 39.1% (45/115) in the CAU group. However, the difference was not statistically significant (OR 1.3, 95% CI 0.7-2.3, *P*=.39).

**Table 3:** Participants with clinical significant improvement.

Outcome	Intervention, n (%) (n=116)	CAU, n (%) (n=115)	OR (95% CI)	*P* value
CES-D	71 (61.2)	73 (63.5)	0.9 (0.5, 1.6)	.82
HADS	36 (31.0)	24 (20.9)	1.7 (0.9, 3.3)	.11
MBI-exhaustion	34 (29.3)	21 (18.3)	1.9 (1.0, 3.7)	.07
MBI-cynicism	16 (13.8)	13 (11.3)	1.2 (0.5, 3.1)	.67
MBI-reduced professional efficacy	10 (8.6)	12 (10.4)	0.8 (0.3, 2.2)	.61

## Discussion

### Principal Results

This study examined the effects of a Web-based guided self-help course, Happy@Work, on depressive symptoms (primary outcome), anxiety symptoms, 3 burnout dimensions (exhaustion, cynicism, reduced professional efficacy), and work performance (secondary outcomes) compared to CAU in employees with depressive symptoms. The study did not corroborate evidence for the effectiveness of the Web-based course compared to CAU in reducing depressive symptoms. Depressive symptoms had improved substantially in both groups at posttreatment with approximately 62% of participants showing a clinically significant improvement in both conditions. Small but significant effects in favor of the intervention group were found on 2 secondary outcomes: anxiety symptoms (*d*=.16) and the burnout dimension exhaustion (*d*=.17). However, the number of people that showed a clinically significant improvement on these measures at posttreatment did not differ between both groups. We did not find additional gains of the intervention on the other outcomes cynicism, reduced professional efficacy, and work performance.

### Comparison With Prior Work

The results regarding depression are not in-line with the overall positive effects of Web-based interventions that are often found when they are compared to nonactive control groups [21,22,61]. Within-group improvement in depression in the intervention condition is comparable with other studies that examined the effects of Web-based PST in the general population [38,62]. However, these studies did not show such large improvements on depression in the control group. Furthermore, these studies showed equal improvement scores on the CES-D, but baseline scores were higher compared to this study.

Several reasons may explain the large reduction in depressive symptoms in the control group. First, an explanation could be that participants in the CAU group showed spontaneous recovery, a phenomenon which is seen frequently in patients with depression and stress [62,63]. It could be possible that spontaneous recovery is higher among those who are still at work while they experience depressive symptoms. It is known from previous research that work-related aspects are of importance in the recovery of depression [64], but to our knowledge there is no research available that subscribes that work-related aspects are related to spontaneous recovery. Second, it could be possible that our method of recruitment resulted in a selection of highly motivated employees who were willing to change. This could have let to improvement by itself.

Third, being able to function and stay at work while experiencing depressive symptoms might have had a positive influence on recovery of depressive symptoms [24,63,65]. Social support from colleagues and supervisors, social identity, regular activities, and time structure are all reported as positive effects of work on mental health [66]. Fourth, the introduction of this study in the participating companies may have had beneficial effects. A company’s participation in this study gives a positive and caring signal to all employees. This may create a more open environment within the company and it might, therefore, be possible that several participants in the CAU group discussed their mental health problems with their supervisor. This may have resulted in recovery of depressive symptoms. Finally, participants in the CAU group received an email with the randomization outcome in which they were also informed about their level of depressive symptoms and they were advised to seek treatment or help for their complaints. This email could have instigated a behavioral change in such a way that participants in the CAU group moved from the preparation stage to the action stage, according to the stages-of-change model from Prochaska and colleagues [67]. Only a small percentage of the participants in the CAU group sought help in professional health care. However, it could be possible that other participants sought help in a different way; for example, via their significant other or via other self-help treatments.

Between-group significant effects were found on 2 of the secondary outcomes in this study: anxiety symptoms and exhaustion (one of the dimensions of burnout). However, these results were not confirmed by clinically significant improvement scores, which may have to do with a lack of power. It is remarkable that significant effects on anxiety symptoms but not on depressive symptoms were found because anxiety and depression often co-occur [68]. The between-group effect sizes for anxiety and depression were similar, but only the effect size on anxiety symptoms was significant. This may indicate that improvement on anxiety symptoms is more stable for all participants. Not many studies examine burnout as a secondary outcome in studies on depression treatment. However, when burnout was assessed in intervention studies with a focus on employees with stress or mental health problems, results were often conflicting. Some studies found positive results on dimensions of burnout and others have not [69,70]. This study only showed significant effect on the exhaustion dimension of burnout. This dimension is sometimes seen as the core dimension of burnout and best reflects the subject’s incapacity to act, primarily because of fatigue [71,72], which is also a symptom of depression. The other 2 dimensions, cynicism and reduced professional efficacy, reflect the subject’s willingness to act, which indicates a certain attitude or cognition [72]. Therefore, it may be possible to find effects in the long term instead of directly after treatment because it is more difficult to change an attitude in a short period of time. This result also indicates the importance of a specific focus on work-related aspects, stress, and burnout symptoms within the intervention. However, interpretation of the positive results on anxiety symptoms and exhaustion needs to be done with care because these effects could have occurred by chance and the effect sizes were very small.

### Limitations

This study has several limitations. The first has to do with the response rate and missing data handling. We were confronted with a high attrition rate which is seen more often in Web-based interventions [73,74]. Attrition was significantly higher in the intervention group, but we could not find baseline differences between the groups (except for country of birth) to identify possible selection bias. The bias that still may have been introduced was accounted for by applying multiple imputation techniques. Nevertheless, imputing 26% of the data may have led to unreliable estimates.

Second, completion of the intervention in this study was low compared with several other studies [38,62,75,76]. Only 26.7% of the participants completed the entire course within 7 weeks, and 38.8% completed lesson 5 within 7 weeks. Therefore, our analysis of improvement scores in the subgroup of course completers has a lack of power. If the course completion was higher, the higher effect size (*d*=0.29) for depressive symptoms may possibly have been significant. The low completion rate of the intervention is highly influenced by the fact that participants only received 7 weeks to complete the intervention due to the study setting. Most participants were simply not able to complete more lessons within 7 weeks and only a few participants stopped the intervention at their own request due to lack of time or other reasons. We sent several email reminders to increase completion rates, but it may have been beneficial to use other methods as well, such as telephone support in addition to Web-based support [77], increased use of persuasive technology elements [78], or tailored feedback [79]. However, it is not yet clear what methods are effective in reducing dropout of Web-based interventions [74]. Third, the participants in this study were primarily Dutch white-collar workers with high educational levels. Therefore, it is uncertain whether the results can be generalized to the general working population. It is also possible that we only recruited employees with high job security. Because of an economic recession at the time of study, recruitment job security was low for many employees. It may have been possible that employees with depressive symptoms who were experiencing low job security did not apply for this study because they were afraid for their privacy. Finally, although the use of a diagnostic interview is a great strength of this study, we did not assess the CIDI interview at posttreatment; therefore, we do not know how many patients met the criteria for major depressive disorder at posttreatment.

### Implications and Future Research

The results of this study implicate that the intervention Happy@Work is not more effective in reducing depressive symptoms than CAU immediately after the intervention. All participants improved substantially between the 2 assessments on depressive symptoms and significant effects in favor of the intervention group were found on anxiety symptoms and emotional exhaustion. Several explanations may account for the high improvement rates in the CAU group. More research is needed to examine the possibilities of using e-mental health in the worksite setting and future research should further explore the needs of employees with mental health problems. Definitive conclusions about the effectiveness of the intervention can be made once long-term effects of the intervention are known.

### Conclusions

This study gives a first impression of the short-term effects of a Web-based guided self-help course for employees with depressive symptoms who are not on sick leave. High within-group effect sizes for both the intervention and the CAU group were found for depressive symptoms. Statistically significant between-group effects in favor of the intervention were found for anxiety symptoms and the exhaustion dimension of the MBI, but with small effect sizes. Clinical significant improvement and reliable recovery effects did not show any significant effects in favor of the intervention. Long-term effects of this intervention need to be studied, including the role of possible mediators and moderators, as well as the cost-effectiveness of the intervention.

## Multimedia Appendix 1

Screenshots from the participant view on the platform.

## Multimedia Appendix 2

CONSORT-EHEALTH checklist V1.6.2 [80].

## Abbreviations

CAU: care as usual
CES-D: Center for Epidemiological Studies Depression scale
CIDI: Composite International Diagnostic Interview
HADS: Hospital Anxiety and Depression Scale
HPQ: Health and Work Performance Questionnaire
ICC: intraclass correlation
ITT: intention-to-treat
MBI: Maslach Burnout Inventory-General Scale
PST: problem-solving treatment
