# Supplementary material for: Short-Term Effects of a Web-Based Guided Self-Help Intervention for Employees With Depressive Symptoms: Randomized Controlled Trial
Source: J Med Internet Res. 2014 May 6;16(5):e121. doi: 10.2196/jmir.3185 (PMC4026573; doi:10.2196/jmir.3185)
Supplement: Supplementary file 2 [file jmir_v16i5e121_app2.pdf]

CONSORT-EHEALTH Checklist V1.6.2 Report

(based on CONSORT-EHEALTH V1.6), available at [http://tinyurl.com/consort-ehealth-v1-6].

3185

Date completed

12/12/2013 9:41:50

by

Anna Geraedts

Web-based guided self-help for employees with depressive symptoms: short-term effects of a randomized controlled trial

TITLE

1a-i) Identify the mode of delivery in the title

'Web-based guided self-help for employees with depressive symptoms: short-term effects of a randomized controlled trial"

1a-ii) Non-web-based components or important co-interventions in title

No non-web-based components

1a-iii) Primary condition or target group in the title

'Web-based guided self-help for employees with depressive symptoms: short-term effects of a randomized controlled trial"

ABSTRACT

1b-i) Key features/functionalities/components of the intervention and comparator in the METHODS section of the ABSTRACT

'The intervention (Happy@Work) is based on problem solving treatment and cognitive therapy and consistsed of 6 weekly lessons. Participants were asked to submit their weekly assignment via the website after completion and subsequently they received feedback from a coach via the website."

1b-ii) Level of human involvement in the METHODS section of the ABSTRACT

'Participants were asked to submit their weekly assignment via the website after completion and subsequently they received feedback from a coach via the website."

1b-iii) Open vs. closed, web-based (self-assessment) vs. face-to-face assessments in the METHODS section of the ABSTRACT

'We recruited employees from 6 different companies via the company's intranet and posters. "

1b-iv) RESULTS section in abstract must contain use data

'A total of 231 employees were randomized to either the intervention group (n=116) or care-as-usual (n=115).The post-treatment assessment was completed by 171 (74%) subjects. "

1b-v) CONCLUSIONS/DISCUSSION in abstract for negative trials

'This study showed that a web-based guided self-help course for employees with depressive symptoms was not more effective in reducing depressive symptoms among employees than care-as-usual."

INTRODUCTION

2a-i) Problem and the type of system/solution

'Recently, the Organisation for Economic Co-operation and Development [OECD] OECD (2012) published a report in which they concluded that employees with mental health problems are frequently treated when symptoms have become severe and that the work setting of the employee is not often discussed in treatment. They recommend to increase evidence-based workplace treatments for employees with mental health problems and to intervene in an earlier stage, preferably before sick leave. Other research further subscribes the importance of intervening before sick leave, since it will prevent worsening of mental health problems and as a result it will reduce the costs of work absenteeism and loss of work productivity (citation). Unfortunately, there is almost no research available on the treatment of employees with mental health problems who are not on sick leave (citation). The study by Lexis et al. (2011) showed positive results of a face-to-face problem-solving treatment for employees with a high risk for sick leave due to depressive symptoms. The promising results of this study indicate the importance of investment in intervening before sick leave. Providing such a preventive intervention via the internet can have many advantages as previously mentioned.
The current randomized controlled trial evaluated the effectiveness of a web-based guided self-help course for employees with depressive symptoms who were not on sick leave compared to care-as-usual (CAU)."

2a-ii) Scientific background, rationale: What is known about the (type of) system

'Research on the treatment of depression has been extensive (citation) and many studies have shown positive effects of different psychotherapies (citation). Traditionally, most types of psychotherapies are delivered face-to-face in mental health care settings. However, there is increasing evidence for the effectiveness of guided self-help treatments that are delivered via the internet (citation). Web-based treatments generally use the same techniques as face-to-face treatments, but patients can work through the treatment on their own in an often highly structured way. Web-based treatments may reduce costs and can increase efficiency in mental health care because of several advantages, such as high accessibility, no waiting list and minimal contact with a professional therapist (citation). High accessibility may be of special benefit to employees, since they will not lose work hours due to therapist visits outside the workplace and participation in web-based treatments is more anonymous compared to face-to-face treatment. The evidence for effective worker-directed interventions for employees with depressive symptoms is scarce (citation). Some research has been conducted on the treatment of employees who reported sick from work (sick-listed employees) with common mental health disorders and the results of these studies are conflicting (citation). Previous research also shows that only a small percentage of employees with severe mental health problems seek professional treatment (citation). Work related aspects, such as a higher risk of job loss and social support at work, play an important role in the development and perpetuation of depression (citation). Work related aspects, such as high job insecurity, can have a negative effect on symptom severity, and symptom severity can have a negative effect on work elements, such as job performance. It is therefore important to develop evidence based worker-directed interventions for employees with depression which involve work related aspects and the employability of the employee (citation).
"

METHODS

3a) CONSORT: Description of trial design (such as parallel, factorial) including allocation ratio

'The first aim was to examine whether the web-based intervention had a more positive effect on depressive symptoms compared to the CAU group. The second aim was to investigate if the intervention had positive effects on symptoms of anxiety, burnout and work performance."

3b) CONSORT: Important changes to methods after trial commencement (such as eligibility criteria), with reasons

no changes

3b-i) Bug fixes, Downtimes, Content Changes

'There were no content changes, bug fixes, or periods of downtimes required during the trial. "

4a) CONSORT: Eligibility criteria for participants

'Subjects were eligible to take part if they: were 18 years of age or older, had elevated depressive symptoms (scoring 16 or higher on the Center for Epidemiologic Studies Depression – scale [CES-D]), were not on partial or full sick leave, had access to the Internet and an e-mail address, and were employed by one of the six participating companies."

4a-i) Computer / Internet literacy

'had access to the Internet and an e-mail address"

4a-ii) Open vs. closed, web-based vs. face-to-face assessments:
'Subjects were recruited via banners and digital pamphlets on the company's intranet and via posters (only in company 5). Recruitment took place between September 2011 and December 2012. Subjects who were interested in taking part in the study could ask for more information about the study via e-mail. When information was requested, one of the researchers sent an information leaflet and an informed consent form via e-mail. The informed consent could be returned via post or e-mail. After subjects had given informed consent, they received a link to an online screening questionnaire via e-mail. "
"Subjects also participated in a clinical interview at T0 via telephone. "
"The CIDI was conducted by trained interviewers via telephone at baseline (T0) and was used for diagnostic purposes."
4a-iii) Information giving during recruitment

4b) CONSORT: Settings and locations where the data were collected
'Subjects filled in several questionnaires at baseline (T0) and 8 weeks later (T1). Both assessments took place online. "
4b-i) Report if outcomes were (self-)assessed through online questionnaires
'Subjects filled in several questionnaires at baseline (T0) and 8 weeks later (T1). Both assessments took place online. "
4b-ii) Report how institutional affiliations are displayed

5) CONSORT: Describe the interventions for each group with sufficient details to allow replication, including how and when they were actually administered
5-i) Mention names, credential, affiliations of the developers, sponsors, and owners
'The intervention Happy@Work (https://happy-at-work.e-behandeling.nl/) is a brief web-based intervention delivered with minimal guidance. It was developed by the authors for this study "
5-ii) Describe the history/development process

5-iii) Revisions and updating
'There were no content changes, bug fixes, or periods of downtimes required during the trial. "
5-iv) Quality assurance methods

5-v) Ensure replicability by publishing the source code, and/or providing screenshots/screen-capture video, and/or providing flowcharts of the algorithms used
'Screenshots of the intervention can be found in figure 1 and Multimedia Appendix 1. "
5-vi) Digital preservation
'The intervention Happy@Work (https://happy-at-work.e-behandeling.nl/) is a brief web-based intervention"
5-vii) Access
'At the start of the intervention, an account was generated by the researchers on the website and a coach was assigned to the participant on the website. Once the account was generated, an automatic e-mail was sent to the participant with a link to activate the account. Participants used their e-mail address and a self-created password to log in once the account was activated. "
5-viii) Mode of delivery, features/functionalities/components of the intervention and comparator, and the theoretical framework
'In PST, it is assumed that depressive symptoms can be caused by practical problems that people face in their daily lives. It is believed that, when people can resolve their problems, their symptoms of depression will decrease [36]. The PST will help them solve their problems. Sometimes, however, problem solving can be disrupted by automatic thoughts such as ''I am too weak to solve this problem'' or ''I will fail solving this problem''. PST may not be sufficient to change these automatic thoughts that disrupt problem solving. Therefore, we incorporated CT information and assignments to change these automatic thoughts in the course"
"The course intervention consists of 6 weekly lessons with an option of one week extra time in case of delay. Each lesson has a different theme, but always follows the same structure: information about the theme, examples, and assignments. Themes of the lessons are: introduction of problem solving (lesson 1), problem solving methods (lesson 2), changing cognitions (lesson 3), dealing with work related problems (lesson 4), social support (lesson 5), and relapse prevention (lesson 6). "
"Participants were asked to submit their weekly assignment via the website after completion and subsequently they received feedback from a coach, again via the website, within three working days. Next, automatic e-mails were sent to participants to notify them about the feedback, to describe the following lesson, and to give the deadline for completion of the next assignment. Participants were able to start with a new lesson after they had received the feedback (i.e. tunnelled intervention)."
5-ix) Describe use parameters
'Participants were asked to submit their weekly assignment via the website after completion and subsequently they received feedback from a coach, again via the website, within three working days. "
5-x) Clarify the level of human involvement
'Participants were asked to submit their weekly assignment via the website after completion and subsequently they received feedback from a coach, again via the website, within three working days. "
"The coaches were Master level students in clinical psychology that had followed a training of six hours. All coaches used a protocol-treatment manual throughout the course. To ensure treatment fidelity, all feedback was reviewed by a supervisor (AG) before it was placed on the website. "
5-xi) Report any prompts/reminders used
'Participants were asked to submit their weekly assignment via the website after completion and subsequently they received feedback from a coach, again via the website, within three working days. Next, automatic e-mails were sent to participants to notify them about the feedback, to describe the following lesson, and to give the deadline for completion of the next assignment. Participants were able to start with a new lesson after they had received the feedback (i.e. tunnelled intervention). When deadlines were not met, e-mail reminders were sent to participants. "
5-xii) Describe any co-interventions (incl. training/support)
'Subjects in both conditions had access to any additional (mental) health care. "
6a) CONSORT: Completely defined pre-specified primary and secondary outcome measures, including how and when they were assessed

'Subjects filled in several questionnaires at baseline (T0) and 8 weeks later (T1). Both assessments took place online. Subjects also participated in a clinical interview at T0 via telephone. "
"Depressive symptoms
Symptoms of depression were measured with the Center for Epidemiological Studies Depression – scale (CES-D; citation). This questionnaire is widely used for identifying people with depressive symptoms. Its validity has been tested in different populations (citation). The CES-D consists of 20 items and the total score varies between 0 and 60. A score of 16 or higher represents a clinically significant level of depressive symptoms (citation). The cut-off score of 16 was used in this study as an inclusion criterion.
Anxiety symptoms
The anxiety subscale of the Hospital Anxiety and Depression Scale (HADS) was used to measure anxiety symptoms (citation). The anxiety subscale of the HADS consists of 7 items. Scores range from 0 to 21, with higher scores indicating more anxiety. The HADS has shown good homogeneity and reliability in different normal and clinical Dutch samples (citation).
Burnout symptoms
Burnout symptoms were measured with the Dutch version of the Maslach Burnout Inventory-General Scale (MBI; citation). This self-report questionnaire contains 15 items and three dimensions: Exhaustion (5 items); Cynicism (4 items); reduced Professional Efficacy (6 items). Every item was scored on a 7- point Likert scale (0-6). Following the manual of the questionnaire (citation), a total score for every dimension was calculated by adding the item scores and dividing that total score by the number of items, with higher scores indicating more severe symptoms. Participants with a high score on Exhaustion and a high score on Cynicism or a high score on reduced Professional Efficacy were considered as ‘’burnout’’ (citation). We rescored the Professional Efficacy dimension with higher scores indicating less feeling of professional efficacy, hence the high score in the burnout diagnoses.
Work performance
We used the general work performance scale of the WHO Health and Work Performance Questionnaire (HPQ; citation) which contains four items. Item 4 gives the best and easiest indication of the subject’s perception of their own work performance (citation) and we will only report that item in this study. On item 4 subjects were asked to rate their overall work performance during the past four weeks when compared to employees with comparable functions. It was scored on a 7- point Likert scale with a higher score indicating poorer work performance compared to other employees (citation).
Clinical interview
The World Health Organization Composite International Diagnostic Interview (CIDI, version 2.1; citation) is a structured interview to assess psychiatric diagnosis defined in the Diagnostic and Statistical Manual of the American Psychiatric Association (4th ed., text rev.; DSM-IV-TR; citation). For this study, two sections of the CIDI were assessed: the mood disorders section, and the ‘’other’’ anxiety disorders (social phobia, panic disorder, agoraphobia and generalized anxiety disorder) section. The CIDI was conducted by trained interviewers via telephone at baseline (T0) and was used for diagnostic purposes.
Other measures
We added demographic questions, working hours, medication use for psychological problems and treatment by a mental health specialist to the baseline questionnaire. At T0 and T1 we collected data on healthcare utilization by subjects. "

6a-i) Online questionnaires: describe if they were validated for online use and apply CHERRIES items to describe how the questionnaires were designed/deployed

6a-ii) Describe whether and how “use” (including intensity of use/dosage) was defined/measured/monitored

completion of lessons was recorded for every participant and reported in the results.

6a-iii) Describe whether, how, and when qualitative feedback from participants was obtained

Satisfaction with the intervention was assessed at post treatment. This will be reported separately.

6b) CONSORT: Any changes to trial outcomes after the trial commenced, with reasons

no changes.

7a) CONSORT: How sample size was determined

7a-i) Describe whether and how expected attrition was taken into account when calculating the sample size

'The sample size was guided by the expected difference in the primary outcome (i.e. depressive symptoms) between the two groups. Based on a power of 0.80, an alpha of 0.05, and an expected drop-out percentage of 30%, we would need 100 subjects in each condition to be able to show an effect-size Cohen’s d of 0.50. Therefore, the total sample size was determined at 200. "

7b) CONSORT: When applicable, explanation of any interim analyses and stopping guidelines

not applicable

8a) CONSORT: Method used to generate the random allocation sequence

'Block randomization was used with random blocks containing 4, 6 or 8 allocations. An independent researcher made the allocation schedule with a computerized random number generator and the investigators had no knowledge of the schedule. "

8b) CONSORT: Type of randomisation; details of any restriction (such as blocking and block size)

'Block randomization was used with random blocks containing 4, 6 or 8 allocations. An independent researcher made the allocation schedule with a computerized random number generator and the investigators had no knowledge of the schedule. "

9) CONSORT: Mechanism used to implement the random allocation sequence (such as sequentially numbered containers), describing any steps taken to conceal the sequence until interventions were assigned

'Block randomization was used with random blocks containing 4, 6 or 8 allocations. An independent researcher made the allocation schedule with a computerized random number generator and the investigators had no knowledge of the schedule. "

10) CONSORT: Who generated the random allocation sequence, who enrolled participants, and who assigned participants to interventions

'Block randomization was used with random blocks containing 4, 6 or 8 allocations. An independent researcher made the allocation schedule with a computerized random number generator and the investigators had no knowledge of the schedule. "

11a) CONSORT: Blinding - If done, who was blinded after assignment to interventions (for example, participants, care providers, those assessing outcomes) and how

11a-i) Specify who was blinded, and who wasn’t

participants and researchers were not blinded (not possible).
"Subjects were randomized into two groups: the intervention group or the care-as-usual (CAU) group. Subjects were informed about the randomization outcome via e-mail. "

11a-ii) Discuss e.g., whether participants knew which intervention was the “intervention of interest” and which one was the “comparator”

'Subjects were randomized into two groups: the intervention group or the care-as-usual (CAU) group. Subjects were informed about the randomization outcome via e-mail. "

11b) CONSORT: If relevant, description of the similarity of interventions

not similar.

12a) CONSORT: Statistical methods used to compare groups for primary and secondary outcomes

'Baseline differences in demographic variables and outcome measures were investigated using Chi-square tests and independent-sample t-tests. "

12a-i) Imputation techniques to deal with attrition / missing values

'Baseline data were available for all subjects. Missing values at post-treatment (T1) (26%, 171/231) were handled by multiple imputation using the Fully Conditioned Specified Method (FCSM) with model type predictive mean matching (PMM) in SPSS creating five datasets. In multiple imputation, missing data are imputed by regression analyses and the available baseline data (demographics and baseline scores on outcome measurements) of the study completers as well as the study drop-outs at post-treatment are used to estimate missing values at post-treatment. This provides a more reliable estimation of the ''real'' data than other imputation methods. The analyses were performed on the five created data sets and combined into a single overall estimate using the multiple imputation inference rules of Rubin (1987). This yields proper p values and confidence intervals for the estimates. "

12b) CONSORT: Methods for additional analyses, such as subgroup analyses and adjusted analyses

no additional analyses

RESULTS

13a) CONSORT: For each group, the numbers of participants who were randomly assigned, received intended treatment, and were analysed for the primary outcome

CONSORT flowdiagram included

13b) CONSORT: For each group, losses and exclusions after randomisation, together with reasons

CONSORT flowdiagram included

"Of the 116 participants randomized to the intervention group, 9.5% (n=11) did not start or complete the first lesson of Happy@Work. Lesson 1 was completed by 90.5% (n=105), lesson 2 by 75% (n=87), lesson 3 by 57.8% (n=67), lesson 4 by 49.1% (n=57), lesson 5 by 38.8% (n=45) and lesson 6 by 26.7% (n=32). The majority of the participants who dropped out did not report a reason for drop out. When reasons were reported (n=14) they pertained mostly to lack of time. "

13b-i) Attrition diagram

no diagram, only text:

"Of the 116 participants randomized to the intervention group, 9.5% (n=11) did not start or complete the first lesson of Happy@Work. Lesson 1 was completed by 90.5% (n=105), lesson 2 by 75% (n=87), lesson 3 by 57.8% (n=67), lesson 4 by 49.1% (n=57), lesson 5 by 38.8% (n=45) and lesson 6 by 26.7% (n=32). The majority of the participants who dropped out did not report a reason for drop out. When reasons were reported (n=14) they pertained mostly to lack of time. "

14a) CONSORT: Dates defining the periods of recruitment and follow-up

'Recruitment took place between September 2011 and December 2012. "

"Subjects filled in several questionnaires at baseline (T0) and 8 weeks later (T1). "

14a-i) Indicate if critical “secular events” fell into the study period

none.

14b) CONSORT: Why the trial ended or was stopped (early)

Ended as planned.

15) CONSORT: A table showing baseline demographic and clinical characteristics for each group

Table 1.

15-i) Report demographics associated with digital divide issues

Table 1.

16a) CONSORT: For each group, number of participants (denominator) included in each analysis and whether the analysis was by original assigned groups

16-i) Report multiple “denominators” and provide definitions

reported in text and tables.

16-ii) Primary analysis should be intent-to-treat

yes, ITT used.

17a) CONSORT: For each primary and secondary outcome, results for each group, and the estimated effect size and its precision (such as 95% confidence interval)

yes, reported in text and tables.

17a-i) Presentation of process outcomes such as metrics of use and intensity of use

'Of the 116 participants randomized to the intervention group, 9.5% (n=11) did not start or complete the first lesson of Happy@Work. Lesson 1 was completed by 90.5% (n=105), lesson 2 by 75% (n=87), lesson 3 by 57.8% (n=67), lesson 4 by 49.1% (n=57), lesson 5 by 38.8% (n=45) and lesson 6 by 26.7% (n=32). The majority of the participants who dropped out did not report a reason for drop out. When reasons were reported (n=14) they pertained mostly to lack of time. "

Process evaluation will be reported separately.

17b) CONSORT: For binary outcomes, presentation of both absolute and relative effect sizes is recommended

'Data on clinical significant improvement are reported in Table 3. Clinical significant improvement on depressive symptoms was comparable in both groups (OR = 0.9, 95% CI: 0.5 – 1.6, P = .82). More subjects in the intervention group showed clinical significant improvement on anxiety symptoms, the exhaustion dimension of the MBI, and the cynicism dimension of the MBI compared to the CAU group, but differences between the groups were not significant (see Table 3). A total of 105 subjects (45.5%) were recovered from depression at post-treatment. More subjects in the intervention group (48.3%) recovered from depression compared to the CAU group (42.6%), but not significantly (OR = 1.3, 95% CI: 0.7 – 2.3, P = .41). Reliable recovery rates for depression were also in favor for the intervention group, with an odds ratio of 1.3. In the intervention group 44.8% reliably recovered and 39.1% in the CAU group. However the difference was not statistically significant (OR = 1.3, 95% CI: 0.7 – 2.3, P = .39). "

18) CONSORT: Results of any other analyses performed, including subgroup analyses and adjusted analyses, distinguishing pre-specified from exploratory

'Explorative subgroup analyses were performed to look into more detail whether the intervention was effective for specific subgroups of depression severity but there were no significant results (data not shown). "

18-i) Subgroup analysis of comparing only users

reported outcomes of course completers in text and table 2.

19) CONSORT: All important harms or unintended effects in each group

not apllicable, no harms.

19-i) Include privacy breaches, technical problems

19-ii) Include qualitative feedback from participants or observations from staff/researchers

DISCUSSION

20) CONSORT: Trial limitations, addressing sources of potential bias, imprecision, multiplicity of analyses

20-i) Typical limitations in ehealth trials

'This study has several limitations. The first has to do with the response rate and missing data handling. We were confronted with a high attrition rate which is seen more often in web-based interventions (citation). Attrition was significantly higher in the intervention group, but we could not find baseline differences between the groups (except for country of birth) to identify possible selection bias. The bias that still may have been introduced was accounted for by applying multiple imputation techniques. Nevertheless, imputing 26% of the data may have led to unreliable estimates.
Second, completion of the intervention in this study was low compared to several other studies (citation). Only 26.7% of the participants completed the entire course within 7 weeks, and 38.8% completed lesson 5 within 7 weeks. Therefore, our analysis of improvement scores in the subgroup of course-completers has a lack of power. If the course-completion would have been higher, it might be possible that the higher effect size (d=0.29) for depressive symptoms, would have been significant. Lack of time was the most frequently reported reason for drop-out and completion rates might have been higher if we had not used strict time frames of completing one lesson a week (with one week extra in case of delay). We sent several e-mail reminders to increase completion rates, but it may have been beneficial to use other methods as well such as telephone support in addition to web-based support (citation). However, it is not yet clear what methods are effective in reducing drop-out of web-based interventions (citation). "

21) CONSORT: Generalisability (external validity, applicability) of the trial findings

21-i) Generalizability to other populations

'Finally, the subjects in this study were mainly Dutch white collar workers with a high educational level. It is therefore uncertain whether the results can be generalized to the general working population. It is also possible that we only recruited employees with a high job security. Due to an economic recession at the time of study recruitment job security was low for many employees. It may have been possible that employees with depressive symptoms who were experiencing low job security did not apply for this study because they were afraid for their privacy. "

21-ii) Discuss if there were elements in the RCT that would be different in a routine application setting

this will be reported separately in process evaluation.

22) CONSORT: Interpretation consistent with results, balancing benefits and harms, and considering other relevant evidence

22-i) Restate study questions and summarize the answers suggested by the data, starting with primary outcomes and process outcomes (use)

'This study examined the effects of a web-based guided self-help course ‘Happy@Work’ on depressive symptoms (primary outcome), anxiety symptoms, three burnout dimensions: exhaustion, cynicism, reduced professional efficacy, and work-performance (secondary outcomes) compared to care as usual in employees with depressive symptoms. The study did not corroborate evidence for the effectiveness of the web-based course compared to care-as-usual in reducing depressive symptoms. Depressive symptoms had improved substantially in both groups at post-treatment with about 62% of participants showing a clinically significant improvement in both conditions. Small but significant effects in favor of the intervention group were found on two secondary outcomes, anxiety symptoms (d=.16) and the burnout dimension exhaustion (d=.17). However, the number of people that showed a clinically significant improvement on these measures at post-treatment did not differ between both groups. We did not find additional gains of the intervention on the other outcomes cynicism, reduced professional efficacy, and work performance. "

22-ii) Highlight unanswered new questions, suggest future research

'The results of this study implicate that the intervention Happy@Work is not more effective in reducing depressive symptoms than care-as-usual immediately after the intervention. All subjects improved substantially between the two assessments on depressive symptoms and significant effects in favor of the intervention group were found on anxiety symptoms and emotional exhaustion. Several explanations may account for the high improvement rates in the CAU group. More research is needed to examine the possibilities of using e-mental health in the worksite setting and future research should further explore the needs of employees with mental health problems. Definitive conclusions about the effectiveness of the intervention can be made once long-term effects of the intervention are known. "

Other information

23) CONSORT: Registration number and name of trial registry

Trial registration: Nederlands Trial Register (NTR): NTR2993

24) CONSORT: Where the full trial protocol can be accessed, if available

text contains a reference to the protol article.

25) CONSORT: Sources of funding and other support (such as supply of drugs), role of funders

'This study is funded by Body@Work Research Center for Physical Activity, Work and Health, TNO VUMC, Amsterdam and the EMGO Institute for Health and Care Research, VU University Amsterdam and VU University Medical Center Amsterdam. "

X26-i) Comment on ethics committee approval

'The informed consent could be returned via post or e-mail. After subjects had given informed consent, they received a link to an online screening questionnaire via e-mail. The study protocol, information leaflet, and informed consent form were approved by the Medical Ethics Committee of the VU University Medical Center (registration number 2011/2)."

x26-ii) Outline informed consent procedures

'The informed consent could be returned via post or e-mail. After subjects had given informed consent, they received a link to an online screening questionnaire via e-mail. The study protocol, information leaflet, and informed consent form were approved by the Medical Ethics Committee of the VU University Medical Center (registration number 2011/2)."

X26-iii) Safety and security procedures

X27-i) State the relation of the study team towards the system being evaluated

'The authors declare that they have no competing interests. "
